# COVID-19 presents an opportunity for dental teams to become ready for person-centred care

**DOI:** 10.1038/s41415-021-3715-3

**Published:** 2021-12-10

**Authors:** Koula Asimakopoulou, Ian Mills, Patricia Neville, Sasha Scambler

**Affiliations:** 4141595774001 Reader in Health Psychology, Faculty of Dentistry, Oral and Craniofacial Sciences, King´s College London, UK; 4141595774002 Past Dean, Faculty of General Dental Practice and Associate Professor, Faculty of Health, University of Plymouth, UK; 4141595774003 Lecturer in Social Sciences, Bristol Dental School, University of Bristol, UK; 4141595774004 Reader in Medical Sociology, Faculty of Dentistry, Oral and Craniofacial Sciences, King´s College London, UK

## Abstract

Two publications that recently appeared in the *British Dental Journal *mooted the position that dentistry may not be ready for person-centred care. This commentary takes up this discussion and agrees that while person-centred care is essential to dentistry, it is not without its challenges. Drawing on the multidisciplinary expertise of its authors (two sociologists, one psychologist and a dentist), the nature of these challenges is interrogated further. It also identifies opportunities for change on this position from within the literature, as well as by clinical practice. We present evidence to suggest that the current pandemic has shown that dental teams are capable of rising to a challenge and adapting to change. In this light, we propose that the pandemic presents dental teams with an opportunity to be even more person-centred.

## Key points

- Dental teams have worked hard to adapt to changes brought about by COVID-19 - they are then, by definition, resilient and adaptable.
- COVID-19 has presented dental teams with an opportunity to hone their skills in providing person-centred care.
- Dental teams should be supported in delivering person-centred care during restrictions and beyond.

## Introduction

Person-centred care (PCC) has been a concept that dentists, behavioural scientists working in dentistry and bodies regulating the dental team have been increasingly familiarising themselves with.^1^^,^^2^^,^^3^^,^^4^^,^^5^ There have been numerous definitions of the concept with some of these arising from models explicitly applied to PCC in dental settings.^1^^,^^2^^,^^3^ Key features of most models of PCC involve building a relationship with the patient based on trust, understanding the patient's perspective and context and providing sufficient information and tools to enable shared decision-making, where appropriate. So, PCC is an approach and a model of care where patients are supported to consider treatment options and to make informed choices^6^ within a process that considers patients' views, attitudes and beliefs, rather than just the clinical problem at hand, with due consideration to the complexity of the patient's life.

This complexity is present both anecdotally in clinical practice and in academic writing. Anecdotally, some dental team clinicians feel that, whilst recognising the importance of asking patients open questions, this may prove problematic in terms of focusing the consultation and running to time. Academically, the complexity of PCC was discussed in this journal by Apelian *et al.*^7^ While acknowledging the importance of person-centredness as a philosophy of patient care, their research found that the practice of PCC presented many challenges to dentists. They report that dental teams were generally disinterested in understanding the complexity of patients' lives, unless doing so protected them from litigation or was a means of encouraging the patient to accept a particular treatment option. Dentists contributing to this study were reported to believe that they did not possess the communication skills to engage in holistic conversations about patients' lives, nor did the health system in which they worked provide the opportunity to do so. These views were further supported by an observation that shared decision-making was only really permissible if the patient's decision was going to align with the dentist's recommendation; this finding has been reported previously.^8^^,^^9^ The paper concludes that dentists and dentistry may not be 'ready' for PCC and attributes much of this to the persistence of seeing dentistry as an 'art' and the hegemony of a biomedical/disease-focused approach to dental education.

Their remedy for the conspicuous absence of the 'patient' from dentistry is to 'teach' social and behavioural sciences to dental students. However, Neville^10^ in their letter to the editor is critical of this solution. While the social and behavioural sciences offer much by way of illuminating the psychosocial realities of patients in all their diversity, the proposition that 'adding on' social and behavioural sciences to dental curricula automatically contributes to a shift in the values and ethos of PCC is, they argue, naive at best. Dental, educational research reveals the existence (and persistence) of a 'hidden curriculum' when healthcare and the patient experience are explored from a psychological and social perspective. Implicit and explicit knowledge hierarchies within dentistry means that dentistry and dental education prioritise biomedical knowledge over social science knowledge.^11^^,^^12^ This hidden curriculum disadvantages prospective dentists and their patients because it means that 'disease' and 'curing' are prioritised over 'health' and 'helping'. As a result, dentist and patient needs can be in direct opposition. We know that patients value the mutual respect and understanding that a person-centred consultation offers^13^^,^^14^ and at the same time we know that dentists often struggle with the concept of PCC.^2^ How do we reconcile such competing orientations to PCC? Although not a view directly suggested by Apelian* et al.,*^7^ one might ask whether a struggle with a concept is sufficient reason to consider abandoning promotion of PCC in dentistry, when it is simply based on the premise that dentists may just be 'not ready' for change.

We propose that the reported situation in Quebec^7^ may not necessarily accord with the UK experience. In the UK, changes in the undergraduate curriculum^15^ and an increasing focus on patient-reported experience measures within the NHS,^16^ as well as consumerism within the private sector,^17^ have placed PCC at the heart of consultations. Further, Apelian *et al.*^7^ refer to 'a rapidly changing society that is losing its confidence in the profession' and although dentistry faces many challenges within the UK, particularly at the present time, a loss of trust or confidence in the dental profession by the public does not appear to be one of them.^14^^,^^18^

We therefore contend that steady progress is already being made in the UK within the PCC agenda and we must continue to develop this further. Any suggestion of abandoning or reducing our commitment to PCC in dentistry, based on an assumption that dentists may not be ready for change, would be inappropriate and inadvisable, in our view. To this end, we present evidence to show that the thinking behind a reduction in emphasis on PCC is flawed idealistically, practically and in relation to professionalism.

## The idealism side of the argument

In terms of idealism, we would invite readers to imagine that a novel technique was invented for bonding crowns or bridges. Imagine the technique was more efficient and patients preferred it but the evidence base for the treatment was only recently available. Next, imagine that the standard-setting bodies in dentistry endorsed this treatment as 'best practice'. Would it be conceivable that dentists would not be expected to use it because 'they were not ready for it'? Of course not. On the contrary, where a novel technique required new technical skills, we would advocate and promote training of the dental team to ensure utilisation of this new technique proficiently and quickly. In fact, 'continuous improvement' is central to dental professionalism^19^ and inherent in the requirement for ongoing continuing professional development (CPD). It is deemed unprofessional for dentists not to update themselves on how to engage with new techniques and skills. Active engagement in CPD has a number of key drivers, which may include fulfilment of regulatory requirements or acquisition of new skills and knowledge, in order to advance personal and professional development. The drivers may be related to compliance, personal ambition, professional fulfilment or peer engagement. There is also a commercial driver which is likely to be particularly relevant within general dental practice, where adoption of the latest technology and advances in dentistry can be used to promote the practice as 'cutting-edge' within the competitive world of high street, private dentistry.

We do wonder then why PCC is not afforded a similar reaction as to a novel dentistry technique? Why is PCC considered too tricky to deliver, when any new dental procedure is likely to be as difficult to learn? The suggestion that PCC is not adopted because dentists are somehow incapable of learning new skills goes against their training and purpose and is risible.

## The practical side of the argument

From a practical point of view, we argue that the idea that dentists are somehow not ready for change also goes against the current evidence. The COVID-19 pandemic and the impact this has had on how dental teams work has been unprecedented. Human society and dentistry have had to rapidly adapt to the unfolding humanitarian and health crisis. For dentistry, COVID-19 has presented a succession of challenges, from the closure of services in the first UK lockdown (March 2020), to the much politicalised scramble for appropriate personal protective equipment (PPE) and the rapid assessment of risk of aerosol generating procedures to ensure the safety of patients and staff.^20^^,^^21^^,^^22^^,^^23^ Dental teams have had to engage with and respond to substantial worries about delivering dentistry during a pandemic.^24^ These challenges have resulted in a massive backlog in treatments and thrown into sharp focus the uneven distribution of NHS dental services and the barriers of access to dentistry for many groups^25^^,^^26^ in the UK. While these structural issues pre-date COVID-19, the current crisis of patient access has put the issue of patient care firmly back on the agenda. NHS dentistry is destined to undergo significant change in the immediate future and the dental profession will undoubtedly be asked to adapt their working practices and philosophies and embrace new ways of working.

## The professionalism side of the argument

Dentists function in an increasingly consumerist environment with a focus on patient feedback and customer care. It would therefore seem implausible that the concept of PCC would not resonate with the profession when the benefits for patients, clinicians and the entire dental team, including reducing the chance of patient litigation, are so apparent. The recent lockdowns of dental services created a particular challenge in terms of professional delivery of care, with a shift to remote triage and a prioritisation of urgent and emergency care. There has been a return to face-to-face care, but access is limited, demand remains high and a significant level of anxiety continues to exist in relation to the pandemic. NHS England have promoted the continued use of remote consultation to address some of these concerns. The dental profession have actively and professionally embraced this opportunity, transferring our new-found penchant for video conferencing into the dental clinic to support remote patient engagement.^27^ While before the COVID-19 pandemic the idea of delivering dentistry online was still in its infancy and the dental profession may have been deemed slow to embrace existing digital technology to allow remote consultation, there is now emerging evidence^27^ that dental teams and patients alike have embraced online consultations. The likelihood is that this trend will continue, even after the risk from COVID-19 is removed.

## Person-centred care after the pandemic - an opportunity to grow

In this type of triage situation, what does PCC even look like? And can we deliver PCC in a time of upheaval and uncertainty? We maintain that while this current crisis presents an array of challenges to the delivery of care, it becomes even more important that the principles of PCC are put into practice. Even during these extremely challenging times for dentistry our patients want and demand their needs be addressed. A failure to look beyond the symptoms of the person in need of care risks storing up even more problems for the future. Patients and members of the dental team have been under intolerable stress and pressure and in such circumstances, tolerance can be low and sensitivities high. Empathy, understanding, connection and communication are key and we would assert that PCC has never been more important than it is at the present time.

Current evidence suggests that dental teams have risen to the challenge presented by the pandemic. Indeed, despite the stress and pressure of the past year, they appear to have the physical and psychological capability to take on pandemic-initiated changes and adapt their practice to ensure that it is COVID-19 compliant.^24^ Dental teams have had to make changes to the care they provide, the way they communicate and how they interact with patients. Some preliminary evidence suggests that despite concerns about online dentistry, dental teams have taken on the task and excelled at it, positively impacting patient satisfaction and allaying patient concerns.^27^

Given then that dental teams have lived up to the challenge of delivering dentistry during a pandemic, what more evidence would one need before concluding that the idea that dentistry 'is an art'^7^ that '…focuses on manual skills and technical procedures that may not favour person-centredness'^7^ is perhaps unsupported? Dental teams seem perfectly capable of moving from a 'techne'^7^ to delivering care through discussion and from a distance.^27^ Dental teams have risen to the practical challenge of COVID-19 with drive and determination and the key values of PCC have been critical factors in the restoration of dental services in the UK. While the additional emotional and psychological impact of living through and providing care in a pandemic may affect the dental team's resilience in engaging with patients, a lack of ability to adopt values of PCC is clearly not a reported issue. The dental profession now need to expand this, ensuring members of the public are aware that they can still access care if needed and this can be supported through remote consultations and crucially by adopting a person-centred approach to allay fears and ensure continuity of care. Remote consultation may have limitations in terms of social interaction and engagement, but it can be used effectively to offer choice for patients, efficiency for the dental team and safe care during a pandemic. The additional benefits in relation to environmental sustainability should not be overlooked as we aim to reduce travel^28^ to offset the carbon footprint created by increased usage and disposal of PPE.^29^

Dentistry has faced many challenges during the pandemic and as a consequence has had to adapt and respond to an unprecedented situation. Members of the dental profession have been exposed to high levels of stress which have been financial, emotional and psychological. Despite the difficult circumstances, many positives will emerge, including new ways of working which is hoped will lead to improved patient care and better working conditions for the dental team.

## Conclusion

Albert Einstein is attributed with the quote 'in the midst of every crisis lies an opportunity'. We believe this is an ideal time to embrace PCC as we build for the future and work towards transformational change in dentistry. To do this, PCC should be a central part of dental education, providing an overview of the theoretical and evidence-based underpinnings of the concept, along with practical guidance on how to practise in a person-centred way. Patient safety and clinical effectiveness have come under close scrutiny during the pandemic and will undoubtedly lead to changes across healthcare, including dentistry. We believe patient experience is equally important and now is the time for change.
